# Is the anterior approach still superior to posterior correction in AIS regarding correction, fusion levels and kyphosis when modern posterior systems are used?

**DOI:** 10.1007/s43390-024-00832-z

**Published:** 2024-03-12

**Authors:** Ahmed Hammad, Johanna Eberl, André Wirries, Florian Geiger

**Affiliations:** 1 Spine and Scoliosis Center, Hessing Foundation, Augsburg, Germany; 2 JW Goethe University Hospital, Frankfurt, Germany

**Keywords:** Scoliosis, AIS, Anterior, Posterior, Correction, Fusion levels

## Abstract

**Purpose:**

The aim of our study is to compare anterior and posterior corrections of thoracic (Lenke I) and lumbar (Lenke V) curves when modern posterior pedicle screw systems with vertebral derotation techniques are used. Curves that could not be corrected with both systems were excluded.

**Methods:**

A thoracic group (*N* = 56) of Lenke I AIS patients (18 anterior and 38 posterior) and a lumbar group (*N* = 42) of Lenke V patients (14 anterior and 28 posterior) with similar curves < 65° were identified.

**Results:**

*Thoracic group* The mean postoperative correction (POC) was 68 ± 13.4% in the anterior and 72 ± 10.5% in the posterior group. The postoperative change in thoracic kyphosis was +4° and +5° respectively. The median length of fusion was eight segments in the posterior and seven segments in the anterior groups. In 89% the LIV was EV or shorter in the anterior, and in 71% of the posterior corrections.

*Lumbar group* The mean POC was 75 ± 18.3% (anterior) and 72 ± 8.5% (posterior). The postoperative gain in lumbar lordosis was 0.8° (anterior) and 4° (posterior). The median length of fusion was five segments in both groups and there was no difference in relation of the LIV to the EV.

**Conclusion:**

With modern implants and derotation techniques, the posterior approach can achieve similar coronal correction, apical derotation and thoracic kyphosis with similar length of fusion and better lumbar lordosis restoration.

## Introduction

Choosing a surgical approach, anterior, posterior or combined, in treating AIS has been controversial for a long time. Classically, AIS has been treated by posterior spinal instrumentation and fusion with good postoperative outcomes [1]. However, limited rotational correction, and consequently the need for longer posterior constructs, were the main drawbacks in the early years [1, 2]. To overcome these limitations, anterior surgery has been initially introduced in 1969 by Dwyer et al. [3] and then further refined and modified by Zielke [4]. A main benefit, compared to the posterior systems available at that time, was the better correction with a shorter construct [1]. Less fusion levels and better kyphosis restoration, had been the main arguments for anterior correction. However, most comparative studies on this topic used dorsal systems which are nowadays no longer available, such as CD or Harrington., With these systems, fusion to the stable vertebra, or with later systems to the last substantially touched vertebra (LSTV), was needed [5–7]. Such guidelines were overcome with modern posterior systems enabling a better 3D deformity correction [7, 8] with better apical derotation from posterior [9].

Considering the drawbacks of the anterior approach such as pulmonary complications, risk of pseudarthrosis, reduction of lumbar lordosis and a higher incidence of junctional kyphosis [10]; this superiority has to be critically reevaluated.

The aim of our study is to compare anterior correction of single thoracic (Lenke I) and lumbar (Lenke V) curves to those achieved with modern pedicle screw-based posterior systems. Both, curve correction in the coronal and sagittal planes as well as the length of fusion in relation to the end vertebra (EV) were investigated.

## Materials and methods

### Study design & patient sample

This is a single-center, single-surgeon study. The data of more than 300 AIS patients, that have received a corrective surgery of their thoracic and/or lumbar curves, have been collected prospectively. To get comparable groups only patients which could easily be addressed from anterior and posterior were included. Thus, double curves and more sever curves were excluded. Two groups have been identified: a thoracic group (*N* = 56) of Lenke I patients with preoperative Cobb angle ranging from 45° to 65°, and a lumbar group (*N* = 42) of Lenke V patients with a preoperative Cobb angle ranging from 40° to 60°. Within each group, two further subgroups, anterior and posterior, were identified. Of the thoracic cases 18 had an anterior and 38 a posterior approach. In the lumbar group we had 14 anterior and 28 posterior cases. The follow-up period was at least 2 years for all patients.

### Surgical details & implants

The length of fusion and types of implant/screws used, were recorded.

For all ventral cases the Halm-Zielke double rod instrumentation was used as described by Halm [11]. The spine was addressed from the convex side. For lumbar curves a thoracophrenicotomy, for thoracic curves a double thoracotomy was performed. All patients received a chest tube for at least three days postoperatively and was then removed when it drained less than 100 ml in 12 h. A meticulous release of the disk space and the anterior longitudinal ligament was performed on each level in order to enable correction and bony fusion. A double rod was used in all cases. The correction was performed by the thicker rod, and the thinner rod was used to create lordosis or kyphosis when needed. After resection of the endplates, bone-on-bone fusion could be achieved. Any possible bone voids were filled with autologous bone. No additional cages were used.

Correction was done by differential rod bending, concave translation and convex cantilever. For thoracic curves, correction was started on the concave side to crerate kyphosis. For lumbar curvesthe, the main correction was done from the convex side to enhance lordosis.

All posterior instrumentations were done with the Expedium system (DePuy) as described before [9, 12]. Polyaxial screws were used for most of the vertebrae, Long-tab screws were used at the apex on the concave side and monoaxial or derotation screws on the convex side. In lumbar curves, a combination of monoaxial and polyaxial screws was used with monoaxial screws on the apex of the convex side to support derotation. Correction was done by differential rod bending, concave translation and convex cantilever. In thoracic curves correction was started on the concave side to create kyphosis. In lumbar curves the main correction was done from the convex side to enhance lordosis. Dorsal release with facet joints resection was done in all cases. None of the cases included in the study received Ponte osteotomies. This is usually done in curves > 70!

Both the anterior double rod technique and posterior pedicle screw instrumentation with derotation apply to the most recent standards.

Intraoperative neuromonitoring with motor evoked potentials (MEP) and EMG was used in all cases. All posterior cases received postoperative epidural anesthesia.

### Selection of the upper and lowest instrumented vertebra (LIV)

When determining the extent of fusion, specific criteria have been applied to preoperatively define the lower and upper instrumented vertebra (LIV & UIV) in both groups. A special caution is needed when selecting the LIV, due to higher risk of distal adding-on, with coronal decompensation, and subsequently higher risk of developing degenerative disk disease.

#### Posterior group

- *LIV* was defined to be:
- the stable vertebra in the side-bending X-ray (does not have to be the stable vertebra in the normal AP film)
- the first vertebra distally, below which the disk space opens on both sides in the right/left bending films
- *UIV* was mostly chosen to be the neutral vertebra cranially.

#### Anterior group

Here, an end-to-end instrumentation has been performed in most of the cases, but if the requirements mentioned above were applicable, even shorter fusions were done.

### Radiographic measures

The radiographic outcome measures were thoracic and lumbar Cobb angle, thoracic kyphosis, apical vertebral translation (AVT), apical vertebral rotation according to Raimondi [13], and coronal imbalance with truncal shift defined as the lateral deviation of the C7 plumbline from the central sacral vertical line (CSVL), in millimeters. All these parameters were identified pre- and postoperatively for all cases by an independent observer. Percentage postoperative Correction (POC), preoperative flexibility (PF) and the Cincinnati Correction Index (CCI) which is the percentage of correction in relation to the flexibility [14] were calculated.

### Complications

All surgery related complications were recorded.

### Statistical methods

Data were statistically described in terms of mean ± standard deviation (±SD). Numerical data were tested for the normal assumption using Shapiro–Wilk test. Comparison of numerical variables between the study groups was done using Student t test for independent samples. Two-sided *P* values less than 0.05 was considered statistically significant. IBM SPSS (Statistical Package for the Social Science; IBM Corp, Armonk, NY, USA) release 22 for Microsoft Windows was used for all statistical analyses.

## Results

### Demographic data

A total of 98 patients with moderate scoliosis, of which 32 received an anterior and 66 received a posterior fusion were included in the study. The perioperative and demographic data are presented in Table 1. Apart from the operation time, that was significantly shorter in the posterior group, as well for thoracic and lumbar curves, demographic data showed no statistically significant differences between the groups.

**Table 1:** Demographic data

	All (*N* = 98)	All Thoracic (*N* = 56)	Thoracic		All Lumbar (*N* = 42)	Lumbar	
			Posterior (*N* = 38)	Anterior (*N* = 18)		Posterior (*N* = 28)	Anterior (*N* = 14)
OP-age	16.32 ± 4.6	15.70 ± 3.4	16.08 ± 3.7	14.89 ± 2.3	17.14 ± 5.9	17.0 ± 4.1	17.43 ± 8.5
LOS	9.84 ± 2.24	9.78 ± 2.2	9.16 ± 1.7	11.18 ± 2.5	9.90 ± 2.4	9.96 ± 2.1	9.8 ± 2.9
OP-time	138.8 ± 45.8	136.0 ± 39.6	128.4 ± 33.6	172.8 ± 48.9	142.5 ± 53.6	112 ± 31	120 ± 30
Gender							
Females	84	47 (83.9%)	33 (86.8%)	14 (77.8%)	37 (88.1%)	25 (89.3%)	12 (85.7%)
Males	14	9 (16.1%)	5 (13.2%)	4 (22.2%)	5 (11.9%)	3 (10.7%)	2 (14.3%)

### Length of fusion

**Fig. 1:**
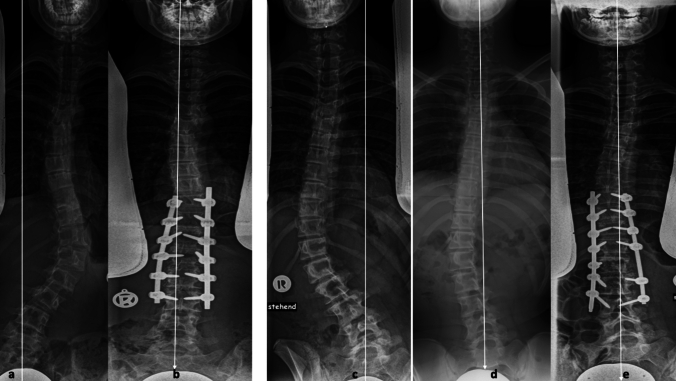
Two examples of posterior correction of thoracolumbar curves. In both cases, the EV was chosen as LIV although it was neither a stable vertebra nor LSTV (**a**, **c**). In the first case, L3 is the lower EV while L4 is the LSTV; T10 is the upper EV (**a**). Fusion was done form T10 (upper EV) to L3 (lower EV—in this case LSTV-1) (**b**). In the second case, L3 is the lower EV while L4 is the LSTV; T8 is the upper EV (**c**). The traction radiograph (**d**) demonstrates that the EV can move in the stable zone; L3 has now become the LSTV (instead of the L4 on the normal AP film). Fusion was done from T9 (EV -1) to L3 (lower EV—in this case LSTV-1 on the normal AP film, but LSTV on the Traction film) (**e**)

**Fig. 2:**
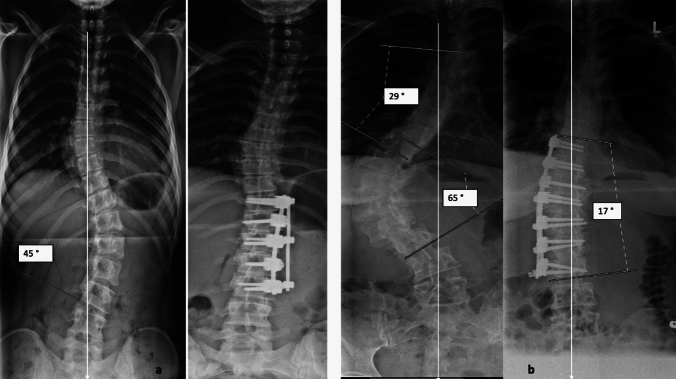
Examples of moderate (**a**) and high grade (**b**) thoracolumbar curves, both instrumented from EV to EV via an anterior double rod system. In the first case **a** fusion was done form T11 (upper EV) to L3 (lower EV & LSTV). In the second case **b** fusion was done from T10 (upper EV) to L3 (lower EV; here LSTV-1)

**Fig. 3:**
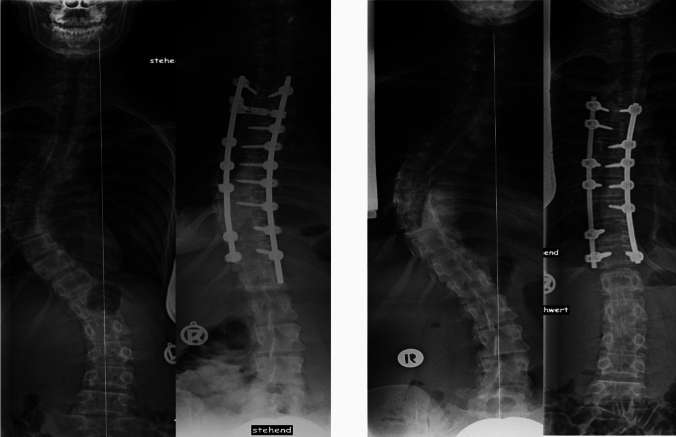
Two examples of single thoracic curves with the EV proximal to the LSTV. In the first case fusion was done from T4 (upper EV) to T12 (lower EV; here being LSTV-1), resulting in a well-compensated spine. In the second case fusion was done from T5 (upper EV) to T12 (EV -1; here being LSTV-2); also resulting in a well-compensated spine

**Fig. 4:**
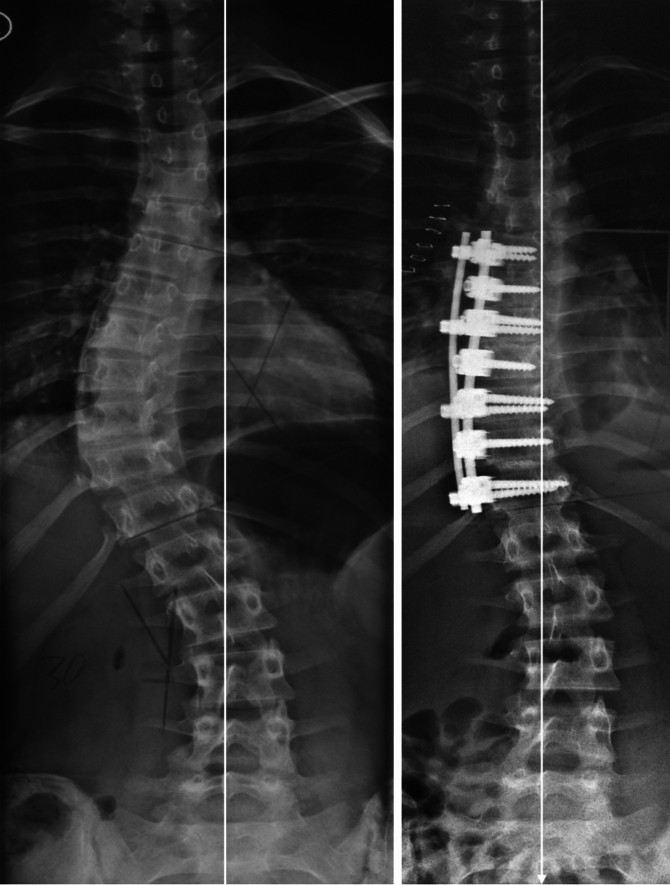
Anterior fusion of a single thoracic curve with a double rod system ending one short of the EV in a 17 years-old basketball player. Fusion was done from T6 (upper EV) to T12 (EV-1; here being LSTV-2)

The length of fusion as well as the LIV, defined in relation to the EV, are presented in Table 2. For lumbar curves a similar length of five segments was fused in both anterior and posterior subgroups. Figures 1 and 2 show examples of end-to-end instrumentation of thoracolumbar/lumbar curves when doing correction posteriorly (Fig. 1) or form anterior (Fig. 2). In thoracic cases, a fusion of eight segments in average was recorded in the posterior and seven segments in the anterior subgroups. The difference was mainly in the UIV which was usually T4 in posterior but T5 in anterior fusion, as T4 can rarely be reached anteriorly because of the overlying vessels. Figures 3 and 4 demonstrate examples of posterior correction (Fig. 3) and anterior fusion (Fig. 4) of thoracic curves.

**Table 2:** Length of fusion (median) & LIV

	Thoracic (*N* = 56)		Lumbar (*N* = 42)	
	Posterior (*N* = 38)	Anterior (*N* = 18)	Posterior (*N* = 28)	Anterior (*N* = 14)
Length of fusion	8	7	5	5
LIV				
EV	22 (58%)	12 (66%)	19 (68%)	9 (64%)
EV−1	5 (13%)	4 (22%)	9 (32%)	4 (29%)
EV+1	11 (29%)	2 (11%)	0 (0%)	1 (7%)

The LIV has been the EV in a comparable portion of cases for posterior and anterior fusions.Differences were not statistically significant neither for thoracic nor for lumbar fusions.

For lumbar curves, of the 28 posterior cases the LIV was L3 in 22 cases (79%) and even L2 in 6 (21%) cases. Of the 22 cases in which we stopped at L3, L3 was the EV in 19 and EV-1 in 3 cases.

Of the 14 anterior cases we stopped at L3 in 10 (71%) and at L2 in 4 (29%) cases. Of the ten cases with L3 as LIV, L3 was the EV in nine cases and EV-1 in 1 case.

#### Coronal correction

*Thoracic curves* The mean preoperative Cobb angle of the major curve was 53.4° (±6) in the posterior and 52.6° (±6.7) in the anterior group; and was corrected to 16.0° (±5.3) and 17.2° (±8.1), respectively (Table 3).

**Table 3:** Pre- & postoperative coronal & sagittal parameters—thoracic group (*N* = 56)

	Mean ± SD	Posterior (*N* = 38)	Anterior (*N* = 18)	*P* value
Preoperative Cobb	53.1 ± 6.2	53.4 ± 6.0	52.6 ± 6.7	0.671
Preoperative flexibility (PF)	39.2 ± 18.6	38.7 ± 20.2	40.0 ± 16.4	0.829
Postoperative Cobb	16.4 ± 6.3	16.0 ± 5.3	17.2 ± 8.1	0.513
Cobb 2-years postop		20.3 ± 6.6	19.3 ± 7.2	
POC %	70.7 ± 11.7	72.3 ± 10.5	68.0 ± 13.4	0.230
CCI (POC/PF)	2.8 ± 5.8	3.2 ± 7.4	2.1 ± 1.2	0.549
Preoperative rotation	19.9 ± 8.7	18.4 ± 8.6	22.9 ± 8.5	0.071
Post operative rotation	9.0 ± 7.1	7.1 ± 6.4	13.1 ± 7.1	0.002*
Deviation from CSVL (preop.)	0.24 ± 15.7	−1.6 ± 15.5	4.1 ± 15.9	0.216
Deviation from CSVL (postop.)	−3.4 ± 18.8	−6.1 ± 17.0	2.4 ± 21.4	0.151
Deviation form CSVL 2-years postop		−4.2 ± 10.2	2.1 ± 18.2	
AVT (preop.)	33.0 ± 20.8	32.0 ± 21.6	35.2 ± 19.6	0.584
AVT (postop.)	0.2 ± 12.4	1.4 ± 12.0	−2.2 ± 13.1	0.332
Preoperative kyphosis	25.6 ± 11.8	25.3 ± 10.9	26.4 ± 14.0	0.761
Postoperative kyphosis	30.2 ± 8.1	30.4 ± 7.7	29.7 ± 9.1	0.790
Change in kyphosis	4.5 ± 9.3	5 ± 7.4	3 ± 12.6	0.378

All significant values (*P* < 0.05) were marked with asterisk symbol

There was no statistically significant difference in PF between the posterior (38.7 ± 20.2) and the anterior (40.0 ± 16.4) corrections. The percentage of operative correction (POC) was slightly better after posterior (72 ± 10.5%) compared to anterior instrumentation (68 ± 13.4%); however, the difference was not statistically significant. Although, there was no significant difference in the preoperative apical rotation (18.4 ± 8.6 in the posterior group and 22.9 ± 8.5 in the anterior group), the postoperative apical rotation was less after posterior surgery (7.1 ± 6.4) than from anterior (13.1 ± 7.1); this difference was statistically significant.

The preoperative deviation from CSVL was 1.6 mm to the left in the posterior, and 4.1 mm to the right in the anterior groups (difference of 2.4 mm—statistically insignificant). This coronal imbalance has worsened slightly postoperatively in the posterior group with 6.1 mm deviation to the left, compared to a slight improvement to 2.4 mm deviation to the right in the anterior group (difference of 3.7 mm—statistically insignificant).

Two years postoperatively, there was a slight increase in the Cobb angle of the main curve (about 4° in the posterior and 2° in the anterior subgroups); however, this slight increase was not statistically or clinically significant and within range of measurement. On the other hand, the overall coronal balance, expressed in the deviation from CSVL, showed a slight spontaneous improvement in both posterior (1.9 mm) and anterior (0.3 mm) cases.

*Lumbar curves* The mean preoperative Cobb angle of the major curve was 48.5° (±5.9) in the posterior group and 48.7° (±6.1) in the anterior group; and was corrected to 13.2° (±5.0) and 11.7° (±8.1), respectively (Table 4). There was no statistically significant difference in flexibility between patients operated from posterior (64.3 ± 12.9%) or anterior (57.1 ± 22.0%). The percentage of correction (POC) was slightly better in the anterior group (75.5 ± 18.2%) compared to posterior (71.8 ± 8.5%). Although there was no significant difference in the preoperative apical rotation (23.4 ± 7.6 in the posterior and 23.8 ± 5.4 in the anterior group), the postoperative apical rotation was significantly less after posterior instrumentation (9.0 ± 6.9) than after anterior (14.0 ± 8.0).

**Table 4:** Pre- & postoperative coronal & sagittal parameters—lumbar group (*N* = 42)

	Mean ± SD	Posterior (*N* = 28)	Anterior (*N* = 14)	*P* value
Preoperative Cobb	48.6 ± 5.9	48.5 ± 5.9	48.7 ± 6.1	0.928
Preoperative flexibility (PF)	61.7 ± 16.8	64.3 ± 12.9	57.1 ± 22.0	0.209
Postoperative Cobb	12.7 ± 6.1	13.2 ± 5.0	11.7 ± 8.1	0.463
Cobb 2-years postop		17.1 ± 7.0	13.5 ± 8.4	
POC %	73.1 ± 12.8	71.8 ± 8.5	75.5 ± 18.2	0.387
CCI (POC/PF)	1.3 ± 0.4	1.2 ± 0.2	1.5 ± 0.6	0.015*
Preoperative rotation	23.5 ± 6.9	23.4 ± 7.6	23.8 ± 5.4	0.835
Post operative rotation	10.6 ± 7.5	9.0 ± 6.9	14.0 ± 8.0	0.046*
Deviation from CSVL (preop.)	−2.3 ± 22.8	−12.1 ± 17.1	15.3 ± 21.6	0.001*
Deviation from CSVL (postop.)	−4.9 ± 19.2	−10.9 ± 15.4	6.6 ± 21.0	0.012*
Deviation form CSVL 2-years postop		−11.6 ± 11.6	5.9 ± 17.1	
AVT (preop.)	−20.8 ± 42.9	−19.0 ± 43.5	−24.0 ± 43.3	0.727
AVT (postop.)	−6.4 ± 14.9	−4.7 ± 13.8	−9.3 ± 16.9	
Preoperative kyphosis	29.7 ± 17.2	29.8 ± 19.7	29.6 ± 11.7	0.983
Postoperative kyphosis	32.3 ± 12.9	32.6 ± 14.1	31.7 ± 10.4	0.829
Change in kyphosis	3.0 ± 8.7	3 ± 9.8	2 ± 6.3	0.616
Preoperative lumbar lordosis	47.0 ± 10.3	49.5 ± 10.7	42.4 ± 7.6	0.018*
Postoperative lumbar lordosis	44.0 ± 8.1	45.3 ± 8.9	41.6 ± 5.7	0.111
Change in lumbar lordosis	−3 ± 2.2	−4.2 ± 1.8	−0.8 ± 1.9	0.001*

All significant values (*P* < 0.05) were marked with asterisk symbol

A deviation of 12.1 mm to the left from CSVL in the posterior compared to 15.3 mm deviation to the right in the anterior groups have been seen. This has improved in both anterior and posterior cases postoperatively. The pre- & postoperative differences between the anterior and posterior subgroups within the lumbar group were statistically significant.

Two years postoperatively, there was only a slight increase of the Cobb angle of the major curve in both subgroups (3.9° posteriorly and 1.8° anteriorly), however with a stable coronal balance.

#### Sagittal correction

*Thoracic group* We could not find a statistically significant differences in pre- or postoperative kyphosis between posterior and anterior patients (Table 3). The postoperative gain in thoracic kyphosis was slightly higher in posterior (5° ± 7.4) than in the anterior (3° ± 12.6) surgeries; however, the difference was not statistically significant.

*Lumbar group* The mean postoperative change in lumbar lordosis was −4° (±1.8) in posterior and −0.8 (±1.9) in anterior operated patients. (Table 4). The spontaneous postoperative change in thoracic kyphosis was slightly higher in the posterior (3° ± 9.8) than in the anterior (2° ± 6.3) group; however, not statistically significant.

### Complications

Within the thoracic group complications included one posterior case with distal adding-on which required revision extending the fusion distally, one wound infection with the need for a revision surgery after posterior surgery. Among the anterior cases, one case of wound healing disturbance was seen, two cases developed a proximal adding-on but required no revision.

In the lumbar group one case of wound healing disturbance and three cases of distal adding-on, requiring extending the fusion distally, were seen in the posterior subgroup; in the anterior subgroup no complications were recorded.

## Discussion

We could show that, using modern instrumentation, comparable short fusions can be achieved from an anterior and posterior approach in moderate single curves. The old dogma that posterior fusions always have to be longer than anterior is no longer valid for the majority of cases if modern pedicle screw-based systems are used. If the disk below the EV opens on both sides in the right/left bending films, and the curve is flexible enough a posterior approach is sufficient. This includes the majority of lumbar curves. For more severe curves anterior release might be beneficial.

Short fusions have predominant clinical importance for the lumbar spine as the number of free segments affects mobility and the risk of developing adjacent degenerative disk disease. As each preserved motion segment counts, we always tried to stay as short as possible especially in lumbar curves. We could achieve a similar length of fusion of five segments using end-to-end instrumentation in 68% from posterior, compared to 64% when doing anterior correction. In about 30% of our cases, we could even stay shorter than the EV. This problem of kyphosis in lumbar curves was addressed with a double rod system. In this way the lordosis could be maintained but not increased significantly.

On the other hand, three posterior cases within the lumbar group (10% of the lumbar cases treated posteriorly) developed distal adding-on requiring revision. L3 was the LIV in two cases (EV-1 in both) and in 1 case L2 was chosen as the LIV (EV-1). The three cases were revised extending the fusion 1 segment distally to the EV. If these three cases would have been primarily fused to the EV rather than EV-1; then theoretically the LIV would have been the EV in 22 posterior cases (79%) compared to 9 anterior cases (64%) within the lumbar group; this difference would have been significant. This will reflect on our selection of the LIV when correcting lumbar curves posteriorly.

In thoracic curves we also had a comparable mean length of fusion of eight segments in posterior and seven segments in the anterior surgeries. A selective fusion with end-to-end instrumentation could be done in 58% of the posterior and 66% of the anterior corrections. As the impact of an additional segment in the thoracolumbar junction is not as big as in the lumbosacral region; we, as most surgeons, had to weigh the risk of an adding-on against the benefit of preserving one more free segment.

Our results differed to the literature, as most studies reported longer instrumentations when using posterior fusion [15–18]. Abel et al. showed in their cohort of 40 patients that when correcting Lenke V curves posterior fusion had significantly more fused levels than anterior fusion [17]. Franic et al. in their meta-analysis [1] showed that on average three fewer fusion segments are needed when performing anterior correction compared to posterior fusion. This correlates with the results of Halm et al. who showed that the anterior approach allows more selective and shorter fusion [19] compared to posterior, where longer constructs were used [1]. But these authors used different techniques for posterior correction than we did. In their meta-analysis, Franic et al. and colleagues showed that nine different posterior instrumentations were used, with multi-segmented hook-screw instrumentation being the most frequent one, followed by Cotrel-Dubousset (CD) [1]; all of which are systems that are no longer used.

An explanation for the shorter anterior fusion was postulated that the removal of the disks allowed for better derotation combined with restoration of the kyphosis. In those days posterior correction was done mainly by distraction and rod rotation. Therefore, Katwicki et al. [20] showed a better correction of the axial rotation with anterior instrumentation. Likewise Franic et al. [1] in their meta-analysis documented a better rotational correction of 49% with an anterior approach compared to 22% achieved by the posterior instrumentation.

Since then, different techniques for posterior direct vertebral derotation (DVD) were described using special derotation screws, uniplanar or monoaxial screws [9, 10, 21–23].We started early with posterior derotation in combination with Ponte osteotomies [9, 12]. In our technique we achieved a better rotational correction for both thoracic and lumbar curves from posterior.

With modern instrumentation and facet resections we could achieve a similar correction of thoracic (72% from posterior compared to 68% anterior) and lumbar curves (71% compared to 75%, respectively). With former techniques this amount of correction could not always be reached. Franic et al. reported 66% correction using the anterior compared to 61% with the posterior approach [1]; and Li et al. [24] showed 54% anterior correction compared to 55% posterior correction. We think that better correction possibilities enabled shorter fusions and hope that this evolution will proceed.

Another factor in favor for anterior correction in thoracic curves was the better kyphosis restoration [1, 10] as most of the AIS curves are hypokyphotic [1, 25]. Rhee et al. [26] examined 110 AIS patients and showed a significant increase in thoracic kyphosis with anterior versus posterior correction (+ 4° vs. −2° for anterior and posterior approaches, respectively). However, this kyphogenic effect has a negative impact when correcting lumbar curves where lordosis is wanted. With our posterior approach we could achieve an increase in thoracic kyphosis of 5° which was similar to anterior correction with an increase of 3°. In lumbar curves, we could enhance the lordosis by 4° with posterior instrumentation compared to only 0.8° from anterior. Thus, posterior correction could achieve a desirable kyphogenic effect in thoracic curves by correcting from the concave side and a lordogenic effect in lumbar curves by derotation from the convex side.

Former studies presented heterogenous results regarding sagittal plane correction by posterior approach. This can be contributed to the different posterior derotation techniques used. While Urbanski and colleagues [27], and Tsirikos et al. [28] showed a better kyphosis restoration; Kim et al. [29] and Lowenstein et al. [30] showed decrease in thoracic kyphosis. We ourselves showed in a previous study [9] an increase in thoracic kyphosis when rotating around the convex side, and on the other side a minimal decrease in thoracic kyphosis when performing correction from the concave side with derotation screws. Thus, it cannot be generally stated that posterior instrumentation leads to decrease of kyphosis. This rather depends on the technique used.

We found shorter operation times in the posterior group, but this could be attributed to the surgeon’s experience. We are aware that other surgeons who perform more anterior scoliosis corrections could have different operation times. In the meantime, as we perform anterior corrections with Vertebral Body Tethering (VBT), the approaches are much more minimal invasive and operation times are also shorter than 2 h for single thoracic curves.

Furthermore the anterior approach was reported to have adverse effects on the pulmonary function [31–33]. A thoracotomy can cause a decline in pulmonary function 3 months postoperatively, which becomes normal by two years postoperatively, but not better as the preoperative levels [10]. The posterior fusion without rip hump resection does not negatively impact the pulmonary function on the long run; it would rather have a positive impact on the respiratory tests at 2- & 6-year follow up [10, 34]. Other factors which led us favor the posterior approach in the last years were the possibility to apply peridural catheters and the faster recovery in absence of chest tubes.

The availability of different pedicle screw systems, including long-tab, monoaxial, unilateral & derotation screws, enable the surgeon to choose between, or combine, different correction/derotation techniques. This resulted in a better, or at least similar, scoliosis correction in all different planes (coronal, sagittal & rotational correction). From our point of view this provides an advantage of the posterior approach considering the approach-related morbidity and the longer operation times associated with the anterior approach.

## Conclusion

Since the last comparative studies, the posterior systems have advanced dramatically. With modern implants and derotation techniques, the posterior approach can achieve similar apical derotation, thoracic kyphosis and better lumbar lordosis restoration, and similar Cobb angle reduction in AIS patients. Similar length of fusion and the possibility to stop at the EV combined with shorter operation times and lower approach-related morbidity let us favor the posterior over the anterior approach for standard curves. This might change in future, as minimal invasive access devices for the anterior approach with a new generation of instruments and implants, may evolve. Hence, the anterior approach should still be considered as an alternative, as it still has the potential benefits of faster bone healing; and better release of stiffer curves.

Thus, new comparative studies to reevaluate the pros and cons of both approaches would then be needed.

## Data Availability

The data that suuport the findings of this study are available, and can be provided anonymously upon request from the corresponding author.
